# High-Performance FET-Based Dopamine-Sensitive Biosensor Platform Based on SOI Substrate

**DOI:** 10.3390/bios13050516

**Published:** 2023-05-03

**Authors:** Tae-Hwan Hyun, Won-Ju Cho

**Affiliations:** Department of Electronic Materials Engineering, Kwangwoon University, Seoul 139-701, Republic of Korea; gusxod97@kw.ac.kr

**Keywords:** dopamine, ion-sensitive FET, biosensor platform, SOI, dual-gate FET, sensitivity, self-amplification, extended gate

## Abstract

Dopamine is a catecholamine neurotransmitter that plays a significant role in the human central nervous system, even at extremely low concentrations. Several studies have focused on rapid and accurate detection of dopamine levels using field-effect transistor (FET)-based sensors. However, conventional approaches have poor dopamine sensitivity with values <11 mV/log [DA]. Hence, it is necessary to increase the sensitivity of FET-based dopamine sensors. In the present study, we proposed a high-performance dopamine-sensitive biosensor platform based on dual-gate FET on a silicon-on-insulator substrate. This proposed biosensor overcame the limitations of conventional approaches. The biosensor platform consisted of a dual-gate FET transducer unit and a dopamine-sensitive extended gate sensing unit. The capacitive coupling between the top- and bottom-gate of the transducer unit allowed for self-amplification of the dopamine sensitivity, resulting in an increased sensitivity of 373.98 mV/log[DA] from concentrations 10 fM to 1 μM. Therefore, the proposed FET-based dopamine sensor is expected to be widely applied as a highly sensitive and reliable biosensor platform, enabling fast and accurate detection of dopamine levels in various applications such as medical diagnosis and drug development.

## 1. Introduction

Dopamine (DA) is a type of excitatory catecholamine neurotransmitter that plays important roles in various human physiological processes, including hormonal regulation, renal functions, cardiovascular functions, and central nervous system regulation [1,2,3]. It plays a particularly vital role in the brain as it is involved in several critical functions, such as movement, emotional regulation, learning, memory, and addiction [4,5]. DA levels must be maintained for normal brain function and overall physical and mental health. Abnormal DA levels, whether too high or too low, can lead to several physical and neurological illnesses, including Parkinson’s disease and Alzheimer’s disease, as well as psychiatric disorders such as schizophrenia and attention deficit hyperactivity disorder [6,7,8]. Hence, precise detection and monitoring of DA concentrations are crucial for the diagnosis and management of several neurological and psychiatric conditions. This necessitates the development of a high-performance DA sensor, which can significantly improve the diagnosis and treatment of DA-related disorders. As DA is maintained at extremely low levels in the human body, such sensors can be important for the early detection of abnormal DA levels, which may indicate underlying disorders [9,10,11]. Therefore, the accurate detection and monitoring of dopamine levels are of utmost importance for managing various neurological and psychiatric conditions, and the development of high-performance dopamine sensors can significantly improve the diagnosis and treatment of dopamine-related disorders. Field-effect transistor (FET)-type chemical sensors have gained significant attention for rapid and accurate label-free detection with low power consumption and CMOS compatibility [12,13,14]. However, the conventional ion-sensitive FET sensor is prone to chemical damage, limiting its practical application. [15,16]. To address this issue, extended-gate field-effect transistor (EGFET) sensor architecture has been introduced, which features sensing units that are electrically isolated from the discrete transducer [17]. EGFET offers the benefit of interchangeable sensing units without compromising the transducer, thus expanding its utility to a variety of analytes, such as viruses, proteins, immunoglobulins, enzymes, and hormones [18,19,20,21]. However, the commercialization of all FET-type sensors is hindered by their low sensitivity, which prevents the detection of small biomolecule signals [15,16]. To achieve sensitivity higher than the Nernstian limit, the single-gate (SG) structure of FET sensors can be replaced by a capacitively coupled dual-gate (DG) structure [22,23,24,25]. The DG FET-type sensor features two gate electrodes that are capacitively coupled by the capacitance ratio between the top-gate oxide (T_ox_) and bottom-gate oxide (B_ox_). This enables self-amplification of the sensitivity in the FET structure, thereby overcoming the sensitivity limitation without using external amplifier circuits.

In this study, we proposed a high-performance DA-sensitive FET-type biosensor platform that comprises an FET transducer unit with a dual-gate (DG) structure and a sensing unit with an extended-gate structure (EG). The transducer unit, which was fabricated on a silicon-on-insulator (SOI) substrate, enables self-amplification of sensitivity based on the capacitance ratio of the insulators of the two gates. The EG sensing unit was prepared by stacking an ITO conductive layer and SnO_2_ sensing layer on a glass substrate. The electrical characteristics of the FET transducer unit were demonstrated by measuring the transfer and output characteristics. Meanwhile, the typical sensing characteristics, such as sensitivity, hysteresis, and drift effect of the fabricated biosensor platform, were evaluated by performing pH sensing operations with a SnO_2_ sensing membrane. The high-performance sensing operation by self-amplification of sensitivity was also examined by comparing pH sensitivity in SG and DG operation modes. Further, the study aimed to evaluate the practical DA detection using the fabricated biosensor platform. A DA-sensitive membrane was created on the SnO_2_ layer through surface functionalization using APTES, 4-CPBA, EDC, NHS, and MES. We found that the low sensitivity of DA in SG operation mode can be enhanced by up to 17 times through capacitive coupling in DG mode by using different concentrations of DA in a 1× PBS solution. Moreover, by changing the concentration of the buffer solution, we evaluated the enhanced sensing characteristics as the buffer solution concentration decreased.

## 2. Materials and Methods

### 2.1. Materials

The materials used in this study included glass substrates (7059 glass; Corning Inc., Corning, NY, USA), SiO_2_ sputter target (purity ≥99.99%, THIFINE Co. Ltd., Incheon, Republic of Korea), indium tin oxide (ITO) sputter target (purity ≥99.99%, THIFINE Co. Ltd.), SnO_2_ sputter target (purity ≥99.99%, THIFINE Co. Ltd.), 30:1 buffered oxide etchant (BOE; J.T. Baker, Phillipsburg, NJ, USA), phosphosilicate glass (PSG; Filmtronics Inc., Butler, PA, USA), polydimethylsiloxane (PDMS; Sylgard 184 silicon elastomer; Dow corning, Midland, MI, USA), pH buffer solution (Samchun chemical, Pyeongtack, Republic of Korea), ethanol (Samchun chemical), (3-aminopropyl)triethoxysilane (APTES; purity ≥99%, molecular weight = 221.37 g/mol, Sigma-Aldrich, St. Louis, MO, USA), 4-carboxyphenylboronic acid (4-CPBA; molecular weight = 165.94 g/mol, Sigma-Aldrich), N-ethyl-N’-(3-dimethylaminopropyl)carbodiimide (EDC; purity ≥97%, molecular weight = 155.24 g/mol, Sigma-Aldrich), N-hydroxysuccinimide (NHS; purity ≥98%, molecular weight = 115.09 g/mol, Sigma-Aldrich), MES hydrate (purity ≥99.5%, molecular weight = 195.24 g/mol, Sigma-Aldrich), phosphate buffered saline (PBS; pH 7.4, Sigma-Aldrich), deionized water (DI water; conductivity ≤4.3 μS/cm, Sigma-Aldrich), and DA hydrochloride (gene information = ADRB1, molecular weight = 189.64 g/mol, Sigma-Aldrich).

### 2.2. Fabrication of SOI DG FET Transducer Unit

**Figure 1:**
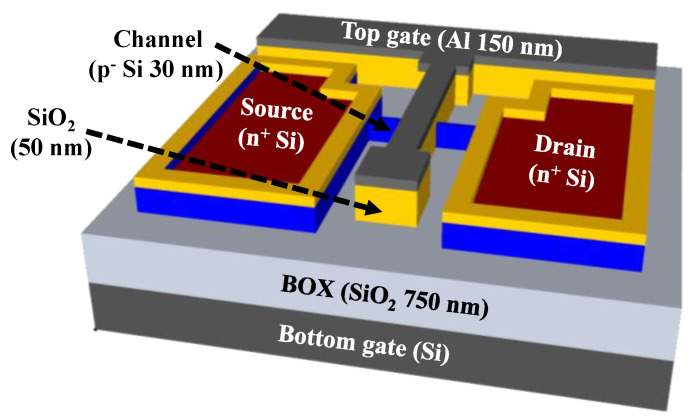
Schematic of the fabricated dual-gate transducer unit on silicon-on-insulator substrate.

The transducer unit used in the present study included two gate electrodes, the top-gate and bottom-gate electrodes, which have different functions to achieve self-amplification of electrochemical signals from biomolecules. The top-gate electrode was connected to the EG sensing unit to receive the electrochemical potential of the biomolecule, while the bottom-gate electrode operated the FET device by applying a gate voltage. Figure 1 illustrates the schematic of the fabricated SOI DG FET transducer unit. p-type (100) SOI substrates of size of 1 × 1 cm^2^ with a top silicon layer (thickness of 30 nm) and a buried oxide (BOX) layer (thickness of 750 nm) were used. The top-silicon layer has resistivity of 1–10 Ω·cm and boron doping concentration of 1 × 10^15^ cm^−3^. The SOI substrate underwent the standard Radio Corporation of America (RCA) cleaning process to eliminate surface impurities and contamination. Active regions with a channel layer were formed by photolithography and a reaction-etching (RIE) process; these regions had width and length of 10 μm and 20 μm, respectively. A 100 nm thick SiO_2_ layer was blanket deposited using RF magnetron sputtering as dummy oxide for the phosphorus doping process. Photolithography process was used for source and drain (S/D) patterning; thereafter, 30:1 BOE was utilized to etch the dummy oxide on the S/D area. For n+ doping of S/D, PSG film was spin-coated and thermally activated using rapid thermal annealing process at 950 °C for 30 s in O_2_/N_2_ ambient. Both the residual PSG and the dummy oxide layer were removed using 30:1 BOE. A 50 nm thick SiO_2_ layer was subsequently deposited as a top-gate oxide using RF magnetron sputtering and photolithography process. The top-gate electrode comprised 150 nm thick Al, which was formed using an electron beam evaporator and lift-off process. To enhance the overall electrical properties of the fabricated transducer unit, a forming gas annealing process was performed at 450 °C for 30 min in 2% H_2_/N_2_ ambient in the furnace system.

### 2.3. Fabrication of DA-Sensitive EG Sensing Unit

**Figure 2:**
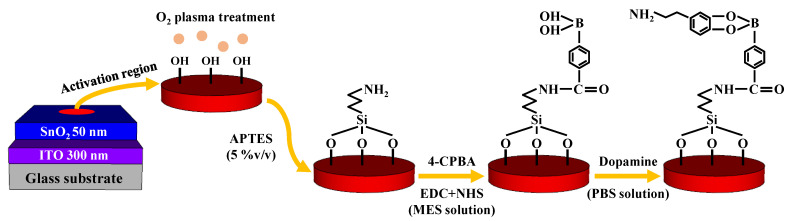
Surface functionalization of DA-sensitive EG sensing unit.

To fabricate the EG sensing unit, a glass substrate (1.5 cm × 2.5 cm) was used. We deposited a 300 nm thick ITO conductive layer and a 50 nm thick SnO_2_ sensing layer using RF magnetron sputtering. The ITO conductive layer was electrically connected to the Al top-gate electrode of the SOI DG FET using an electrical cable. The surface potential of the analytes was sequentially transferred from the SnO_2_ sensing layer to the ITO conductive layer and top-gate electrode of the transducer unit. To function as a DA-sensitive membrane, the SnO_2_ sensing layer underwent several surface functionalization steps. Prior to surface functionalization, we defined an activation region (0.6 cm diameter) on the center of the sensing membrane by attaching a PDMS reservoir. Thereafter, the substrate was subjected to O_2_ plasma treatment for cleaning, which resulted in the formation of OH groups on its surface. Then, we introduced amine groups to the surface using 5% (*v*/*v*) APTES in ethanol solution. The substrate was immersed in the solution at room temperature for 11 min and gently rinsed with ethanol and DI water, after which it was dehydrated in an oven at 70 °C for 1 h to firm the crosslinks on the surface. The substrate was then immersed in a solution of 4-CPBA activated with EDC and NHS in 1 mM MES buffer solution (4-CPBA:EDC:NHS = 1:1:1 mM). We dropcasted 50 μL of 4-CPBA, EDC, and NHS on the surface and kept it at room temperature until all the solution had evaporated. The 4-CPBA acts as a DA receptor since the phenylboronic-acid in it combines with the 1,2- or 1,3-diols of DA and produces reversible covalent bonding [26,27,28]. EDC merges with the carboxyl group of 4-CPBA and forms O-acylisourea [29]. To avoid the hydrolysis reaction that prohibits the amine-carboxyl coupling between the O-acylisourea and amine group, we added NHS solution; NHS attached to the O-acylisourea and produced a stable synthetic NHS ester [30]. After all liquid had evaporated, the activated surface was sequentially rinsed with 10 mM MES buffer solution and DI water. Finally, DA was immobilized on the functionalized surface with dynamic concentrations between 10 fM and 1 μM. DA solutions were prepared by diluting DA hydrochloride in PBS solution (pH 7.4). The process flow of the surface functionalization is depicted in Figure 2.

### 2.4. Device Characterization

The thicknesses of Si, SiO_2_, ITO, and SnO_2_ were measured using a DektakXT Bruker stylus profiler (Bruker, Hamburg, Germany). The electrical characteristics, including transfer and output characteristics of the fabricated SOI DG FET transducers, were measured using an Agilent 4156B precision semiconductor parameter analyzer (Agilent Technologies, Santa Clara, CA, USA). A commercial Ag/AgCl electrode (Horiba 2086A-06T, Kyoto, Japan) was used as the reference electrode to detect DA concentrations and pH buffer solutions. The measurements of the SOI DG FET transducers and DA-sensitive biosensor platforms were carried out in an electromagnetically shielded dark box to exclude external interference such as noise, light, and contamination.

## 3. Results

### 3.1. Electrical Characteristics of SOI DG FET Transducer Unit

**Figure 3:**
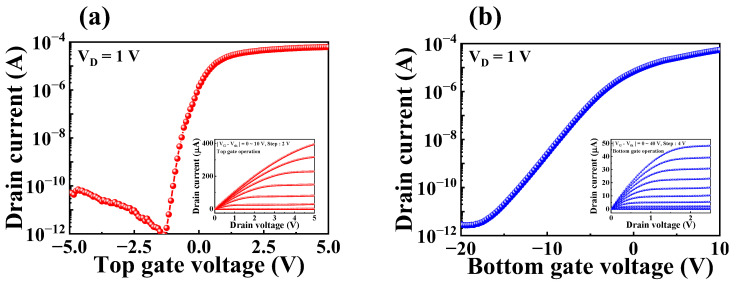
Transfer characteristic curves of the SOI dual gate field-effect transistor (DG FET) transducer with (**a**) top-gate and (**b**) bottom-gate operations. Inset images represent the output characteristic curve for each operation.

The proposed biosensor platform comprised transducer and sensing units. The transducer unit was fabricated on a SOI substrate using a DG structure FET. The sensing performance of the FET-type sensor platform is dependent on the electrical characteristics of the transducer unit. To assess the electrical characteristics of the SOI DG FET transducer unit, transfer and output characteristics were measured. During the electrical characterizations, either the top- or bottom-gate electrode was biased while the other gate electrode was grounded. Figure 3a and b represent the transfer characteristic (I_DS_-V_G_) curves for top-gate and bottom-gate operations of the SOI DG FET transducer device, respectively. These transfer characteristics curves were measured with a drain voltage (V_D_) of 1 V while sweeping the top- and bottom-gate voltage from −5 to 5 V and −20 to 10 V, respectively. The output characteristics (I_DS_-V_D_) for top- and bottom-gate operations—which are shown in the insets of Figure 3a and b—were measured by sweeping V_D_ from 0 to 2.5 V and 0 to 5 V, respectively, while changing the voltages of the two gates from 0 to 40 V and 0 to 10 V, respectively.

Table 1 summarizes the electrical parameters of the fabricated SOI DG FET transducer unit obtained from the transfer characteristic curves. For top-gate operation, the threshold voltage (V_TH_), on/off current ratio (I_ON_/I_OFF_), field-effect mobility (μ_FE_), and subthreshold swing (SS) were −1.1 V, 7.7 × 10^7^, 398.3 cm^2^/V·S, and 135.4 mV/dec, respectively. The corresponding values for the bottom-gate operation were −16.2 V, 1.8 × 10^7^, 98.8 cm^2^/V·S, and 2224.9 mV/dec, respectively.

### 3.2. Self-Amplification through Capacitive Coupling of SOI DG FET Transducer Unit

**Figure 4:**
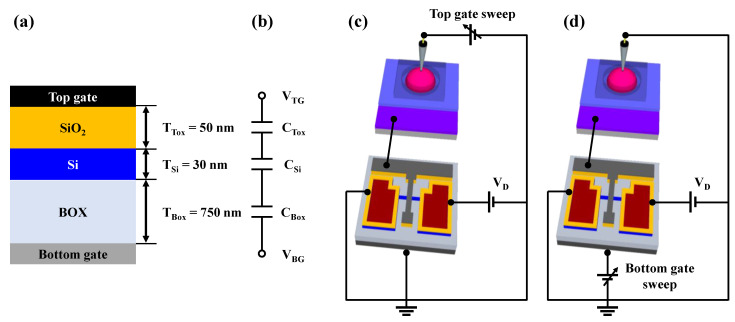
(**a**) Cross-sectional view of metal-oxide-semiconductor capacitor structure and (**b**) equivalent circuit of SOI DG FET transducer unit. Schematic representation of the transducer unit during (**c**) SG mode and (**d**) DG mode.

Figure 4a illustrates the metal-oxide-semiconductor capacitor (MOSCAP) structure, excluding the S/D electrodes, and Figure 4b shows the electrical equivalent circuit of the SOI DG FET transducer unit. In SG operation mode, during which the FET operated using only the top-gate electrode (Figure 4c), sensitivity was not amplified due to the absence of capacitive coupling. However, in DG operation mode, during which the FET operated using the bottom-gate electrode (Figure 4d), sensitivity was amplified based on the capacitance ratio between the top- and bottom-gate electrodes.

Figure 4b and d indicate that the top-gate voltage (V_TG_) and bottom-gate voltage (V_BG_) are capacitively coupled through the top-gate oxide capacitance (C_Tox_) and bottom-gate oxide capacitance (C_Box_). The relationship between V_TG_ and V_BG_ through capacitive coupling can be expressed as Equation (1), which takes into account the depletion region capacitance of the Si channel layer (C_Si_). By expressing Equation (1) in terms of the thicknesses of the top-gate dielectric layer (T_Tox_) and that of the bottom-gate dielectric layer (T_Box_), Equation (2) can be obtained. The depletion region of the Si channel layer has a much thinner equivalent oxide thickness (T_Si_) compared to T_Box_ and T_Tox_. Therefore, T_Si_ can be disregarded, thereby suggesting that the potential applied to V_Tox_ is proportional to the ratio of T_Box_/T_Tox_, as shown in Equation (3). Therefore, the proposed biosensor platform, when operated in DG mode, can detect small potentials by self-amplification using capacitive coupling between the top- and bottom-gate electrodes.

(1) $$∆V_{\mathrm{BG}}=\frac{C_{\mathrm{Si}}C_{\mathrm{Tox}}}{C_{\mathrm{Box}}(C_{\mathrm{Si}}+C_{\mathrm{Tox}})}{∆V}_{\mathrm{TG}}$$

(2) $$∆V_{\mathrm{BG}}=\frac{3T_{\mathrm{Box}}}{3_{\mathrm{Tox}}+T_{\mathrm{Si}}}{∆V}_{\mathrm{TG}}$$

(3) $$∆V_{\mathrm{BG}}\propto \frac{T_{\mathrm{Box}}}{T_{\mathrm{Tox}}}{∆V}_{\mathrm{TG}}$$

The fabricated SOI DG FET had T_Tox_ and T_Box_ of 50 nm and 750 nm, respectively. Hence, the amplification factor of the transducer attained via capacitive coupling was approximately 17 times, allowing for the detection of small biomolecule potentials applied to the EG sensing unit.

### 3.3. pH Sensing Characteristics of the Fabricated FET-Type Biosensor Platform

To validate the practical self-amplification operation and sensing characteristics of the fabricated biosensor platform, we evaluated the pH sensing properties using SnO_2_ as a sensing membrane. The electrochemical sensing mechanism of FET-type sensors was elucidated using the Gouy-Chapman-Stern (GCS) theory and the site-binding model (SBM) [31,32,33]. The GCS theory describes the electrical double layer formed at the interface between the sensing membrane and the electrolyte, whereas the SBM explains the surface reactions occurring between the sensing membrane and the electrolyte. The quantitative relationship according to the potential of the electrical double layer (ψ) in pH sensing operation is described by Equation (4) [34,35]:

(4) $$2.303\left({\mathrm{pH}}_{\mathrm{pzc}}-\mathrm{pH}\right)=\beta \psi \;+\;{\sin h}^{-1}\left[\frac{\sigma_{0}}{2q{\left(K_{b}/K_{a}\right)}^{1/2}N_{s}}\right]-\ln\left(1-\frac{\sigma_{0}}{qN_{s}}\right),$$

where q is the elementary charge, β is the dimensionless chemical sensitivity of the sensing membrane, pH_pzc_ is the pH at which the net charge of the surface is zero, σ_0_ is the charge density, and the N_s_ is the total number of the sites per unit area. The pH_pzc_ and β values of SnO_2_ that we have adopted are 5.6 and 58.6, respectively.

**Figure 5:**
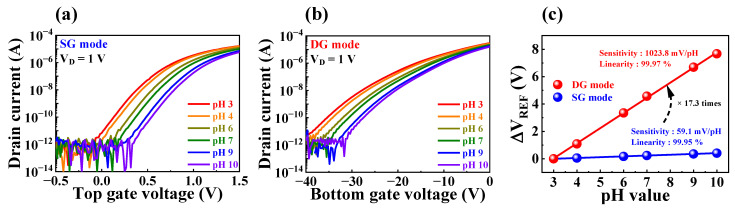
Transfer characteristics curves with varying pH values during (**a**) SG mode and (**b**) DG mode of the proposed biosensor. (**c**) pH sensitivity in the SG and DG modes.

This model was used to determine the sensing characteristics of FET-type sensors using Δψ. However, the conventional single-gate FET-type sensor has a significantly low physical sensitivity limit of 59.14 mV/pH at room temperature, which limits pH sensitivity [15,16]. Nevertheless, incorporating a dual-gate structure into the proposed biosensor platform allows for high-performance detection beyond the physical sensitivity limit through self-amplification via capacitive coupling. Figure 5 presents pH sensing characteristics obtained using the fabricated biosensor. The transfer characteristics curves with varying pH values (ranging between pH 3 and 10) in SG and DG operation modes are shown in Figure 5a and b, respectively. To extract pH sensitivity, the change in reference voltage (V_REF_) based on a current reference (I_R_) of 1 nA for each gate operation was calculated, as shown in Figure 5c. During the SG operation mode, the measured pH sensitivity of 59.1 mV/pH did not surpass the physical sensitivity limit. However, in the DG operation mode, the capacitive coupling between the two gate electrodes resulted in self-amplification of sensitivity, leading to an enhancement in pH sensitivity to approximately 17.3 times higher, with a measured value of 1023.9 mV/pH. The self-amplification capability of the proposed biosensor overcomes the FET-type sensor’s fundamental weakness, enabling more precise detection of biomolecules that are difficult to detect with low potential. Consequently, it was established that the proposed FET-type biosensor platform allowed for high-performance detection operations that are essential for the accurate detection of various biomolecules.

### 3.4. Non-Ideal Effects of the Fabricated FET-Type Biosensor Platform

**Figure 6:**
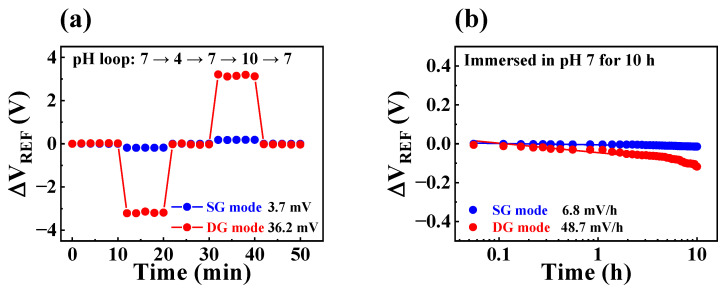
(**a**) Hysteresis effect and (**b**) drift effect in SG and DG modes of the fabricated device.

The sensing membranes in FET-type sensors are vulnerable to chemical damage during sensing operations, which can result in reduced sensing performance. Even when an EGFET structure is employed to protect the FET, the sensing membrane of the EG sensing unit may still be exposed to the different chemicals that are used. Consequently, prolonged, repetitive sensing operations can result in non-ideal phenomena, such as hysteresis and drift effects. Hysteresis effects are commonly caused by the interaction between the surface and electrolyte ions or the gradual transportation of ionic species in the sensing membrane [36,37]. In contrast, the drift effect occurs due to defects in the sensing membrane or permeation of ionic species from the electrolyte through hopping or trap-limited transport [38,39]. To verify the stability of the fabricated FET-type biosensor platform, hysteresis and drift effects were evaluated using a pH solution. In the present study, we conducted hysteresis tests on the SG and DG operation modes for 50 min by adjusting the pH value of the buffer solution in the following pH loop: 7, 4, 7, 10, and 7. The hysteresis voltage (V_H_) was obtained by calculating the V_REF_ difference between the start and end points of the pH loop. Furthermore, we carried out long-term repetitive sensing operations for 10 h while immersed in a pH 7 buffer solution to determine the drift rate (R_D_). Figure 6a and b depict the hysteresis and drift effects of the fabricated device, respectively. The measured V_H_ values in SG and DG operation modes were 3.7 and 36.2 mV, respectively, while the corresponding R_D_ values were 6.8 and 48.2 mV/h, respectively. We observed that capacitive coupling increased the non-ideal effects; in addition, while the pH sensitivity was enhanced by 17.3 times, the V_H_ and R_D_ were limited to only 9.7 and 7.1 times, respectively. These values were within 4.7% of the enhanced sensitivity and did not significantly affect the accuracy of the sensing characteristics. Hence, the sensing characteristics of the fabricated FET-type biosensor platform remained unaffected even after prolonged and repeated measurements. Table 2 summarizes the pH sensing characteristics of the biosensor. The results suggest that the proposed biosensor is a high-performance platform that is capable of stable and reliable sensing operations and has high sensitivity.

### 3.5. DA Sensing Characteristics of the Fabricated DA-Sensitive FET-Type Biosensor

**Figure 7:**
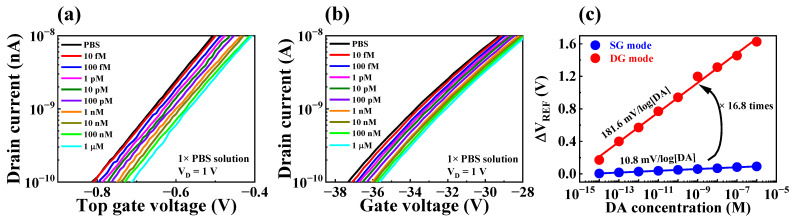
Transfer characteristics curves with varying DA concentration in (**a**) SG mode and (**b**) DG mode. (**c**) DA sensitivity in SG mode and DG mode.

To evaluate the practical utility of the fabricated device as a biosensor, we investigated its sensing performance for DA, a catecholamine that acts as a neurotransmitter in the body and is involved in various functions of the central nervous system [1,2,3]. Detecting the electrochemical potential of DA with precision is challenging due to its extremely low concentration in the body [9,10,11]. However, the fabricated FET-type biosensor platform has the ability to effectively detect DA levels with high sensitivity, owing to its capacity for self-amplification of minute potentials with accuracy. To make the EG-sensing membrane suitable for detecting DA, a sequence of surface functionalization steps was carried out using APTES, 4-CPBA, EDC, NHS, and MES, as illustrated in Figure 2. Additionally, DA hydrochloride was dissolved in 1× PBS (pH 7.4) to prepare DA test solutions with concentrations ranging from 10 fM to 1 μM to immobilize DA onto the DA-sensitive membrane. Following immobilization, the DA sensing characteristics of the fabricated device were assessed at each DA concentration by extracting the ΔV_REF_ at 1 nA of I_R_. The DA sensing characteristics of the DA-sensitive FET-type biosensor are presented in Figure 7. Specifically, Figure 7a and b are the transfer characteristic curves as a function of DA concentration for SG and DG operation modes, respectively. The low DA sensitivity of 10.8 mV/log[DA] measured in SG operation mode increased by about 16.8 times to 181.6 mV/log[DA] through capacitive coupling in the DG operation mode, as shown in Figure 7a. These results suggest that the proposed FET-type DA sensor is capable of effectively detecting DA concentrations and can serve as an efficient biosensor platform that exhibits high sensitivity for detecting small amounts of biomolecules via self-amplification.

**Figure 8:**
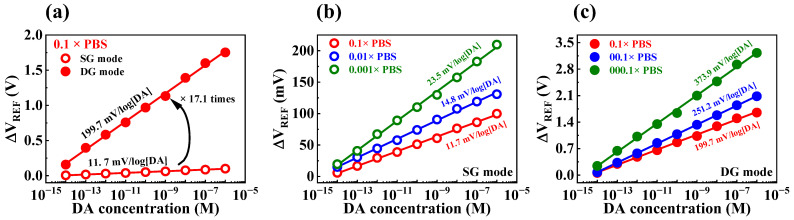
(**a**) DA sensitivity of SOI DG FET transducer with 0.1 M PBS in SG and DG operating modes. DA sensitivity as various PBS concentrations in (**b**) SG and (**c**) DG operating modes.

The DA sensitivities of the fabricated device were determined by exposing the sensor to test solutions containing different concentrations of DA hydrochloride dissolved in PBS solution. However, the PBS buffer solution contains other species, such as KCl, NaCl, Na_2_HPO_4_, and KH_2_PO_4_, which may result in interference; this can impede the transfer of DA potential to the sensing electrode [40,41]. Consequently, the DA sensing characteristics of the sensor could be affected by the shielding effect of ions present in the PBS buffer solution, leading to variations in sensitivity that depend on the buffer concentration. DA sensitivities of the transducer during SG and DG operation modes were 11.7 and 199.7 mV/log[DA], respectively (Figure 8a). Figure 8b and c illustrate the dopamine sensitivities obtained by varying the concentration of PBS in SG and DG operation modes, respectively. In SG operation mode, the dopamine sensitivities were 11.7, 14.8, and 23.5 mV/log[DA] at PBS concentrations of 0.1, 0.01, and 0.001×, respectively. Meanwhile, in DG operation mode, the dopamine sensitivities increased to 199.7, 251.2, and 373.9 mV/log[DA] at the same PBS concentrations. The initial DA sensitivities under SG and DG modes were 10.8 and 181.6 mV/[DA] at 1× PBS solution, which increased to 23.5 and 373.9 mV/log[DA] at 0.001× PBS solution, respectively. This was likely due to a reduction in the shielding effect of ions in the buffer solution as the PBS concentration decreased. Table 3 summarizes the DA sensing characteristics of the fabricated device, showing an extraordinary DA sensitivity of 373.9 mV/log[DA] achieved via self-amplification through capacitive coupling. Thus, we verified that the fabricated DG FET-type biosensor platform is capable of highly sensitive DA sensing using the capacitive coupling effect.

## 4. Conclusions

In this study, we proposed a high-performance biosensor platform for DA detection. This biosensor comprised an FET transducer and EG sensing units. To improve the sensitivity of the sensor, the transducer unit was fabricated using capacitively coupled top- and bottom-gate electrodes on an SOI substrate. We demonstrated that the sensitivity of the transducer can be enhanced by using SnO_2_ as a sensing membrane for pH detection. Using the capacitive coupling of the transducer, a high pH sensitivity of 1023.8 mV/pH was achieved, which exceeded the Nernstian limit. We also evaluated the stability and reliability of the sensor platform by measuring non-ideal behaviors, including hysteresis and drift effect. For biosensor platforms for DA detection, the surface of SnO_2_ was functionalized as a DA-sensitive membrane using APTES, 4-CPBA, EDC, NHS, and MES. We evaluated the DA sensing characteristics by varying DA concentrations in 1× PBS solution. The fabricated device was able to detect low DA concentrations across a concentration range from 10 fM to 1 μM. By operating the sensor in the DG mode, we achieved a 16.8 times increase in DA sensitivity, from 10.8 mV/log[DA] in SG operation mode to 180.6 mV/log[DA]. We further improved the sensitivity by changing the concentration of the buffer solution, resulting in a remarkably high sensitivity of 373.98 mV/log[DA] at 0.001× PBS. Therefore, the proposed dopamine-sensitive FET-type biosensor platform is expected to be a highly applicable, high-performance sensor with stability and reliability, as well as high sensitivity, and could be useful in various biomedical applications, such as the detection of DNA, hormones, antibodies, antigens, enzymes, and viruses.

## Figures and Tables

**Table 1:** Electrical parameters depending on the gate operating mode of the SOI DG FET transducer unit.

Operation Mode	V_TH_ (V)	I_ON/OFF_	μ_FE_ (cm^2^/V·S)	SS (mV/dec)
Top-gate	−1.1	7.7 × 10^7^	398.3	135.4
Bottom-gate	−16.2	1.8 × 10^7^	98.8	2224.9

**Table 2:** pH sensing characteristics of the fabricated FET-type biosensor platform.

Operation Mode	Sensitivity (mV/pH)	V_H_ (mV)	R_D_ (mV/h)	V_H_ to Sensitivity	R_D_ to Sensitivity
SG	59.1	3.7	6.8	6.2%	11%
DG	1023.9	36.2	48.7	3.5%	4.7%

**Table 3:** DA sensing characteristics of the fabricated DA-sensitive biosensor platform.

PBS Concentration	DA Sensitivity (mV/log[DA])		Amplification Ratio
	SG Mode	DG Mode	
1×	10.8	181.6	16.8
0.1×	11.7	199.7	17.1
0.01×	14.8	251.2	16.9
0.001×	23.5	373.9	15.9

## Data Availability

Not applicable.

## References

[1] Franco R., Reyes-Resina I., Navarro G. (2021). Dopamine in health and disease: Much more than a neurotransmitter. Biomedicines.

[2] Basu S., Dasgupta P.S. (2000). Dopamine, a neurotransmitter, influences the immune system. J. Neuroimmunol..

[3] Lin C.-H., Hsiao C.-Y., Hung C.-H., Lo Y.-R., Lee C.-C., Su C.-J., Lin H.-C., Ko F.-H., Huang T.-Y., Yang Y.-S. (2008). Ultrasensitive detection of dopamine using a polysilicon nanowire field-effect transistor. Chem. Commun..

[4] Nieoullon A., Coquerel A. (2003). Dopamine: A key regulator to adapt action, emotion, motivation and cognition. Curr. Opin. Neurol..

[5] Palit S., Singh K., Lou B.-S., Her J.-L., Pang S.-T., Pan T.-M. (2020). Ultrasensitive dopamine detection of indium-zinc oxide on pet flexible based extended-gate field-effect transistor. Sens. Actuator B Chem..

[6] Elsworth J.D., Roth R.H. (1997). Dopamine synthesis, uptake, metabolism, and receptors: Relevance to gene therapy of Parkinson’s disease. Exp. Neurol..

[7] Pan X., Kaminga A.C., Wen S.W., Wu X., Acheampong K., Liu A. (2019). Dopamine and dopamine receptors in Alzheimer’s disease: A systematic review and network meta-analysis. Front. Aging Neurosci..

[8] MacDonald S.W.S., Nyberg L., Bäckman L. (2006). Intra-individual variability in behavior: Links to brain structure, neurotransmission and neuronal activity. Trends Neurosci..

[9] Justice J.B. (1993). Quantitative microdialysis of neurotransmitters. J. Neurosci. Meth..

[10] Smith A.D., Olson R.J., Justice J.B. (1992). Quantitative microdialysis of dopamine in the striatum: Effect of circadian variation. J. Neurosci. Methods.

[11] O’Neill R.D. (1994). Microvoltammetric techniques and sensors for monitoring neurochemical dynamics in vivo: A review. Analyst.

[12] Lee C.-S., Kim S., Kim M. (2009). Ion-sensitive field-effect transistor for biological sensing. Sensors.

[13] Priyadarshani K.N., Singh S., Mohammed M.K.A. (2023). Dielectric/charge density modulated junctionless fet based label-free biosensor. Inorg. Chem. Commun..

[14] Hosseini S.N., Das P.S., Lazarjan V.K., Gagnon-Turcotte G., Bouzid K., Gosselin B. (2023). Recent advances in CMOS electrochemical biosensor design for microbial monitoring: Review and design methodology. IEEE Trans. Biomed. Circuits Syst..

[15] Chen S., Bomer J.G., Carlen E.T., van den Berg A. (2011). Al_2_O_3_/silicon nanoISFET with near ideal Nernstian response. Nano Lett..

[16] Bergveld P. (2003). Thirty years of ISFETOLOGY what happened in the past 30 years and what may happen in the next 30 years. Sen. Actuator B Chem..

[17] van der spiegel J., Lauks I., Chan P., Babic D. (1983). The extended gate chemically sensitive field effect transistor as multi-species microprobe. Sens. Actuator..

[18] Alvarez-Serna B.E., Ramírez-Chavarría R.G., Castillo-Villanueva E., Carrillo-Reyes J., Ramírez-Zamora R.M., Buitrón G., Alvarez-Icaza L. (2023). Label-free and portable field-effect sensor for monitoring rt-lamp products to detect SARS-CoV-2 in wastewater. Talanta.

[19] Hsieh C.-H., Huang C.-H., Lin J.-H., Yu L.-S., Huang I.-Y. (2023). Development of an EGFET microsensor with 3D structure for high-specificity cardiac troponin I detection. J. Micromech. Microeng..

[20] Cho S.-K., Cho W.-J. (2021). Highly sensitive and transparent urea-EnFET based point-of-care diagnostic test sensor with a triple-gate a-IGZO TFT. Sensors.

[21] Chou H.-Y., Chiang J.-L., Yu C.-T.R., Chen J.-M.M., Wuu D.-S. (2023). Sensing property of Ga_2_O_3_-based extended-gate field-effect transistors for a living cell viability sensor. Sens. Actuator A Phys..

[22] Spijkman M.J., Brondijk J.J., Geuns T.C., Smits E.C., Cramer T., Zerbetto F., Stoliar P., Biscarini F., Blom P.W., de Leeuw D.M. (2010). Dual-gate organic field-effect transistors as potentiometric sensors in aqueous solution. Adv. Funct. Mater..

[23] Jang H.-J., Cho W.-J. (2012). Fabrication of high-performance fully depleted silicon-on-insulator based dual-gate ion-sensitive field-effect transistor beyond the Nernstian limit. Appl. Phys. Lett..

[24] Liu N., Hui Liu Y., Feng P., Qiang Zhu L., Shi Y., Wan Q. (2015). Enhancing the pH sensitivity by laterally synergic modulation in dual-gate electric-double-layer transistors. Appl. Phys. Lett..

[25] Cho S.-K., Cho W.-J. (2021). Ultra-high sensitivity pH-sensors using silicon nanowire channel dual-gate field-effect transistors fabricated by electrospun polyvinylpyrrolidone nanofibers pattern template transfer. Sens. Actuator B Chem..

[26] Cambre J.N., Sumerlin B.S. (2011). Biomedical applications of boronic acid polymers. Polymer.

[27] Gu L., Jiang X., Liang Y., Zhou T., Shi G. (2013). Double recognition of dopamine based on a boronic acid functionalized poly(aniline-co-anthranilic acid)–molecularly imprinted polymer composite. Analyst.

[28] Hong S., Lee L.Y.S., So M.-H., Wong K.-Y. (2013). A dopamine electrochemical sensor based on molecularly imprinted poly(acrylamidophenylboronic acid) film. Electroanalysis.

[29] Bartczak D., Kanaras A.G. (2011). Preparation of peptide-functionalized gold nanoparticles using one pot EDC/sulfo-NHS coupling. Langmuir.

[30] Yan Q., Zheng H.-N., Jiang C., Li K., Xiao S.-J. (2015). EDC/NHS activation mechanism of polymethacrylic acid: Anhydride versus NHS-ester. RSC Adv..

[31] Bousse L., De Rooij N.F., Bergveld P. (1983). Operation of chemically sensitive field-effect sensors as a function of the insulator-electrolyte interface. IEEE Trans. Electron. Devices.

[32] Healy T.W. (1974). Site-binding model of the electrical double layer at the oxide/water interface. J. Chem. Soc.-Perkin Trans..

[33] Healy T.W., White L.R. (1978). Ionizable surface group models of aqueous interfaces. Adv. Colloid Interface Sci..

[34] Landheer D., Aers G., McKinnon W.R., Deen M.J., Ranuarez J.C. (2005). Model for the field effect from layers of biological macromolecules on the gates of metal-oxide-semiconductor transistors. J. Appl. Phys..

[35] Majeed L., Amin S.I., Rasool Z., Bashir I., Kumar N., Anand S. (2023). TCAD device modeling and simulation study of organic field effect transistor-based pH sensor with tunable sensitivity for surpassing Nernst limit. Electronics.

[36] Tsai C.-N., Chou J.-C., Sun T.-P., Hsiung S.-K. (2005). Study on the sensing characteristics and hysteresis effect of the tin oxide pH electrode. Sens. Actuator B Chem..

[37] Fung C.D., Cheung P.W., Ko W.H. (1986). A generalized theory of an electrolyte-insulator-semiconductor field-effect transistor. IEEE Trans. Electron. Devices.

[38] Jamasb S., Collins S., Smith R.L. (1998). A physical model for drift in pH ISFETs. Sens. Actuator B Chem..

[39] Bousse L., Bergveld P. (1984). The role of buried OH sites in the response mechanism of inorganic-gate pH-sensitive ISFETs. Sens. Actuator.

[40] Shoute L.C.T., Abdelrasoul G.N., Ma Y., Duarte P.A., Edwards C., Zhuo R., Zeng J., Feng Y., Charlton C.L., Kanji J.N. (2023). Label-free impedimetric immunosensor for point-of-care detection of COVID-19 antibodies. Microsyst. Nanoeng..

[41] Lai P.-H., Tseng L.-S., Yang C.-M., Lu M.S.-C. (2023). Design and characterization of a 16 × 16 CMOS capacitive DNA sensor array. IEEE Sens. J..

